# Recurrent Ogilvie Syndrome as a Possible Manifestation of Alpha-Gal Syndrome (AGS) Requiring Surgical Management: A Case Report

**DOI:** 10.7759/cureus.59357

**Published:** 2024-04-30

**Authors:** Jean Dai, Izi Obokhare

**Affiliations:** 1 General Surgery, Texas Tech University Health Sciences Center (TTUHSC) School of Medicine, Amarillo, USA

**Keywords:** colonic pseudo-obstruction, ogilvie's syndrome, ogilvie, subtotal colectomy with ileostomy, alpha-gal syndrome

## Abstract

We present a case of a male in his 60s with a history of alpha-gal syndrome (AGS) who presented with recurrent acute colonic pseudo-obstruction, also known as Ogilvie syndrome, and underwent surgical treatment for life-limiting symptoms of colonic distention, constipation, and abdominal pain. Prior to surgery, he was hospitalized multiple times after beef consumption and was diagnosed with Ogilvie syndrome, requiring a colonoscopy with rectal tube placement for symptom resolution. He later underwent a robotic subtotal colectomy with ileocolic anastomosis. Follow-up visits showed improvement in symptoms of constipation and abdominal distention. This case highlights that AGS may lead to severe manifestations, such as recurrent Ogilvie syndrome. Due to the increasing prevalence of AGS and limited data on disease course, further research is needed to determine symptom manifestations and the potential utility of surgery in management.

## Introduction

Alpha-gal syndrome (AGS) is a condition in which patients develop an allergy to galactose-α-1,3-galactose, or alpha-gal, a sugar present in the tissues of many non-primate mammals [1]. In the United States, AGS is primarily associated with the lone star tick, or Amblyomma americanum, which inhabits the eastern, southeastern, and central southern states [2]. The lone star tick can transmit alpha-gal to people through its saliva, which can trigger the immune system to produce antibodies against alpha-gal. Affected individuals have allergic reactions when they consume products containing alpha-gal, such as beef, pork, lamb, and other mammal-derived meats [3].

Symptoms of AGS typically manifest as urticaria, nausea, vomiting, constipation, or diarrhea, occurring within three to six hours post-consumption [4]. In severe cases, individuals may experience immediate, life-threatening anaphylactic reactions necessitating urgent medical intervention. Although symptom presentation and remission rates highly vary, some patients can reintroduce meat into their diets without symptom recurrence. Other individuals with AGS may occasionally tolerate red meat but experience mild to severe reactions at other times, which may be influenced by factors like the specific form of alpha-gal in the product or co-factors such as exercise, medications, or alcohol [1].

Alpha-gal is not found in fish, reptiles, or birds [5]. Therefore, some attempts to diagnose AGS were through intradermal skin tests for beef, pork, and milk, with chicken, turkey, and fish serving as negative controls. However, only a few clinics conduct intradermal tests for food antigens, and research shows that these alone are not reliable for a definitive diagnosis [1]. Currently, allergists rely on clinical symptoms, exposure to ticks, and serum levels of IgE to alpha-gal for diagnosis. There are no strict criteria for interpreting serum IgE levels, but the majority of AGS patients have IgE to alpha-gal constituting at least 1% of total IgE for diagnosis [6].

Since 2010, AGS diagnosis has significantly increased. From 2010 to 2018, a study examining AGS geographic distribution in the United States identified more than 34,000 suspected AGS cases in areas known to harbor the lone star tick [7]. The management of AGS patients involves supportive care, symptom control via medication, and dietary modification [1]. It is important to inform patients that further tick bites can either maintain or increase IgE levels to alpha-gal and subsequent allergic reactions. Preventing tick bites in rural or suburban areas can be challenging, but standard precautions like protective clothing and sprays can be effective.

There are currently no reported cases of recurrent Ogilvie syndrome (also known as acute colonic pseudo-obstruction) as a manifestation of AGS. While numerous risk factors contribute to Ogilvie syndrome development, its specific underlying mechanism remains unidentified, and its onset is unpredictable [8]. However, several clinical conditions elevate patient risk, including advanced age, electrolyte imbalance, and immobility. In a retrospective study of 400 patients with Ogilvie syndrome, admission for non-operative trauma, severe infection, and cardiovascular disease each predisposed approximately 10% of cases [9]. Regarding treatment, patients with contraindications to pharmacologic therapy should consider endoscopic decompression. The procedure involves a colonoscopy without gas or bowel prep. If unsuccessful, or if bowel ischemia or perforation occurs, surgery is necessary [8]. In this report, we present a case of refractory and recurrent Ogilvie syndrome with a history of AGS requiring surgical treatment with robotic subtotal colectomy and ileocolic anastomosis. Considering the timing of the patient’s symptoms after the reintroduction of beef to his diet, we presume that AGS may have contributed to his development of refractory Ogilvie syndrome. Due to the recent discovery of AGS, its increasing prevalence, and a wide array of symptoms, further research is needed to determine its potential role in rare manifestations such as Ogilvie syndrome and the potential utility of surgical management in severe cases.

## Case presentation

**Figure 1:**
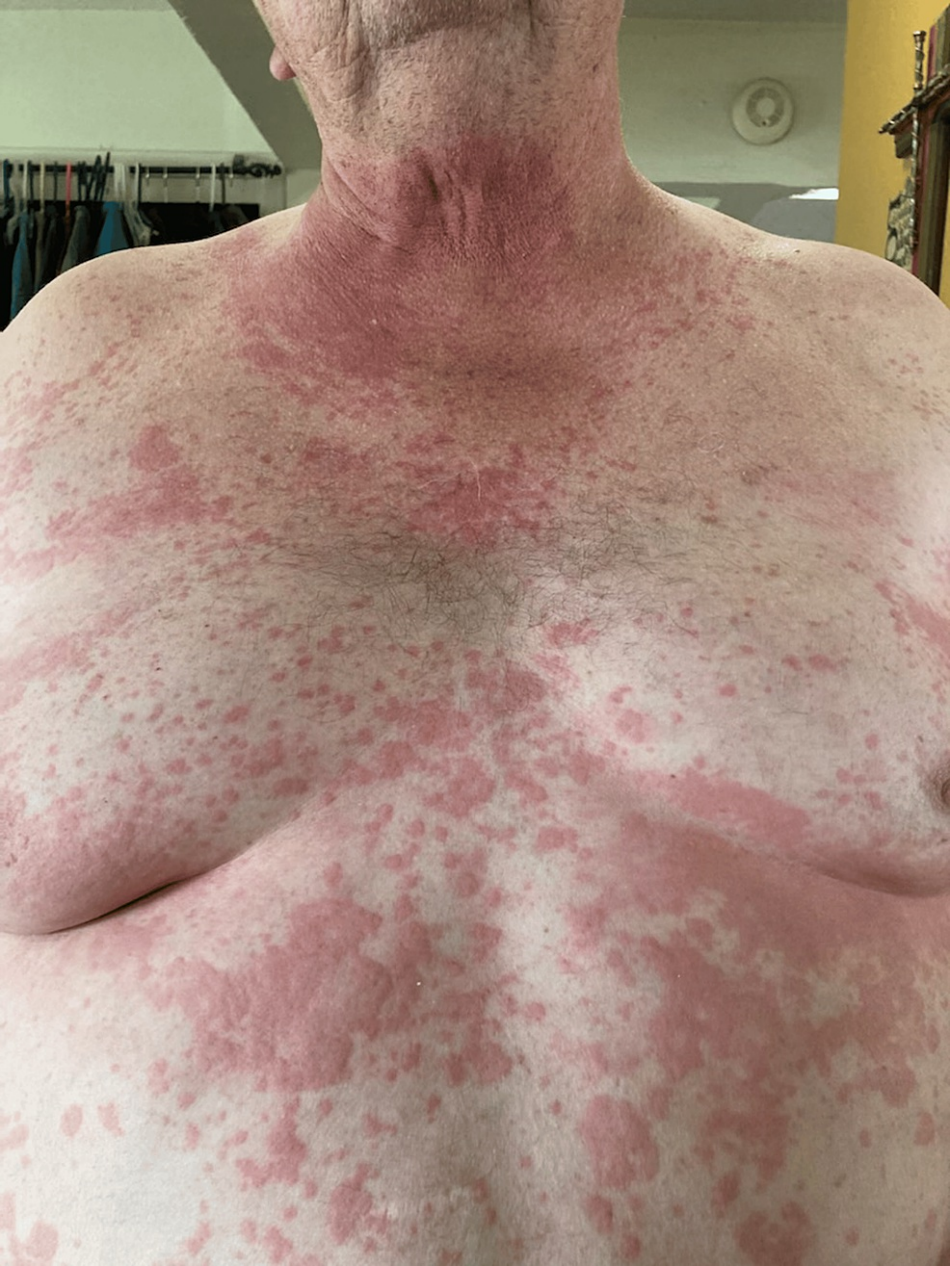
Patient's chest and abdomen within 24 hours post-beef consumption after the diagnosis of alpha-gal syndrome In this image, the patient has widespread urticaria and a maculopapular rash on the chest and abdomen within 24 hours post-beef consumption. The patient approved of the use of the image in this case report.

**Figure 2:**
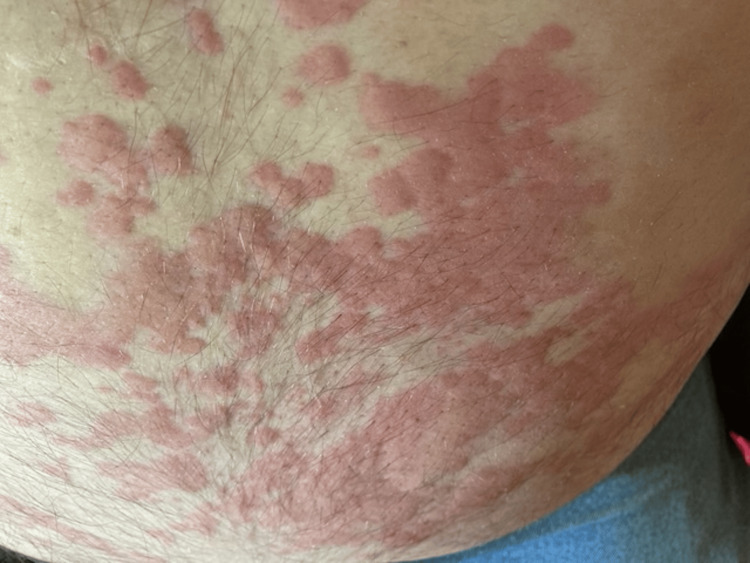
Patient's lower abdomen approximately 24 hours post-beef consumption The image was taken at the same time as Figure 1 shows urticaria on the lower abdomen within 24 hours post-beef consumption. The patient approved of the use of the image in this case report.

The patient was a male in his 60s with a past medical history of obstructive sleep apnea, benign prostatic hyperplasia (BPH), vertigo, left nephrolithiasis, obesity, and venous insufficiency. His family history was negative for cancer, autoimmune disease, or gastrointestinal pathologies. Prior surgical history included a cholecystectomy and a right middle finger distal amputation. He had allergies to shrimp, corn, and penicillin, previously reacting to each with widespread urticaria, swelling, and dyspnea, requiring epinephrine for symptom resolution. About three years prior to surgery, he also developed symptoms of widespread urticaria (Figures 1, 2), constipation, and bloating within 48 hours of mammal meat consumption that would resolve after several days. He was subsequently diagnosed with AGS based on his symptom presentation, exposure to lone star ticks, and positive serum levels of IgE to alpha-gal greater than 1% of total IgE.

**Figure 3:**
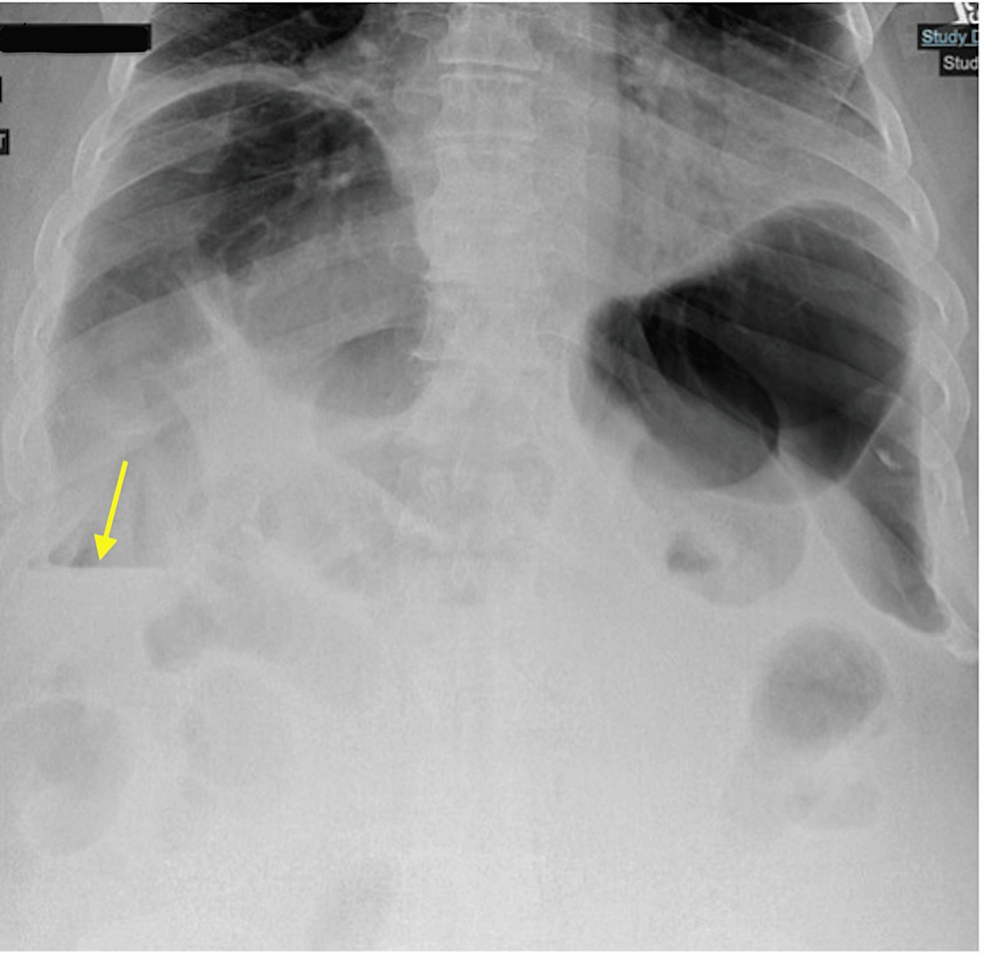
Upright abdominal X-ray An upright abdominal X-ray upon first admission showed colonic dilation, especially in the right and left upper segments, and air-fluid levels in the ascending colon (yellow arrow).

**Figure 4:**
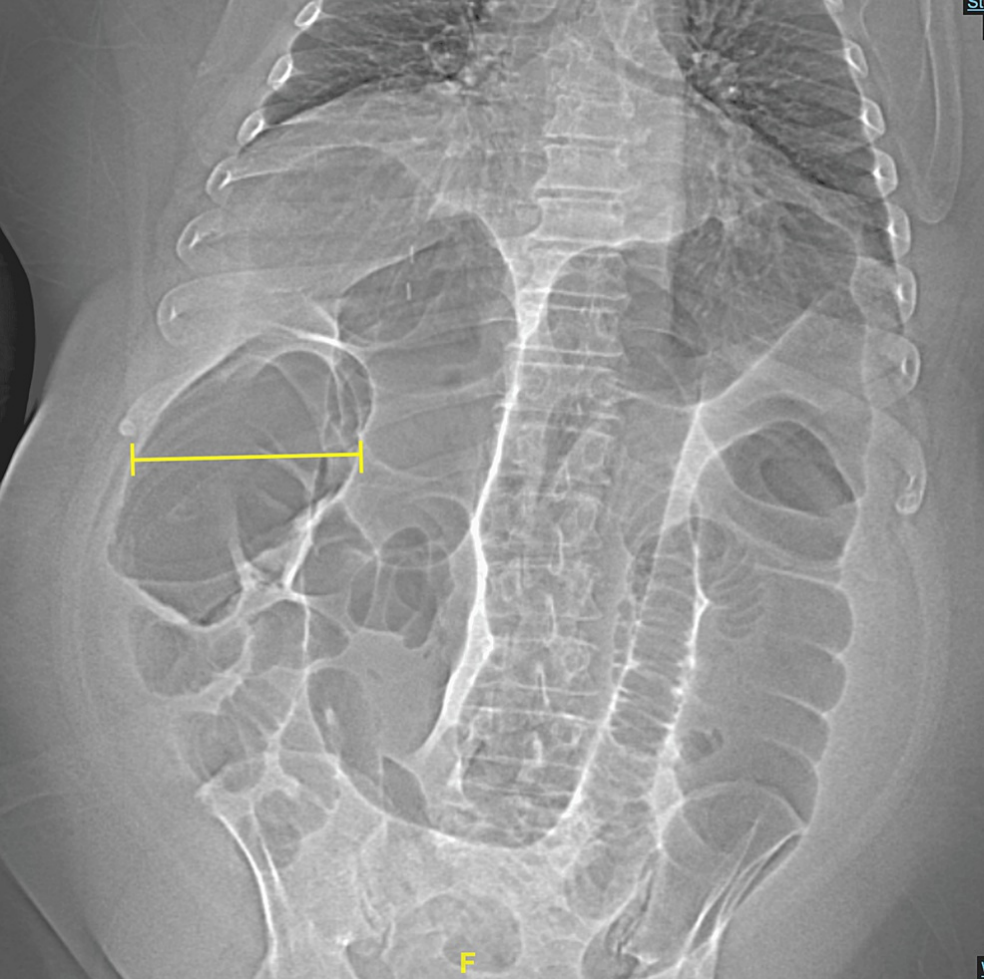
CT abdomen and pelvis topography without contrast Coronal view of CT abdomen and pelvis topography without contrast upon first admission, showing dilation of the ascending colon measuring 11cm (yellow).

After his AGS diagnosis, he eliminated mammal meat and products containing alpha-gal from his diet; however, he was admitted to the hospital twice after attempting to reintroduce beef into his diet. Prior to each admission, he developed urticaria that resolved before presentation to the emergency department. On his first admission, he presented with abdominal distention and a gradual onset of sharp epigastric pain rated 9/10. The pain was intermittent, migrated to the lower abdomen, and was associated with constipation and an inability to orally tolerate solids and liquids. Upright abdominal X-ray (Figure 3) showed significant dilation of the colonic segments in the right upper and left upper quadrants. Computed tomography (CT) of the abdomen and pelvis with rectal contrast demonstrated severe gaseous distention of the colon, especially of the ascending colon, measuring 11 cm in the ascending colon (Figure 4).

**Figure 5:**
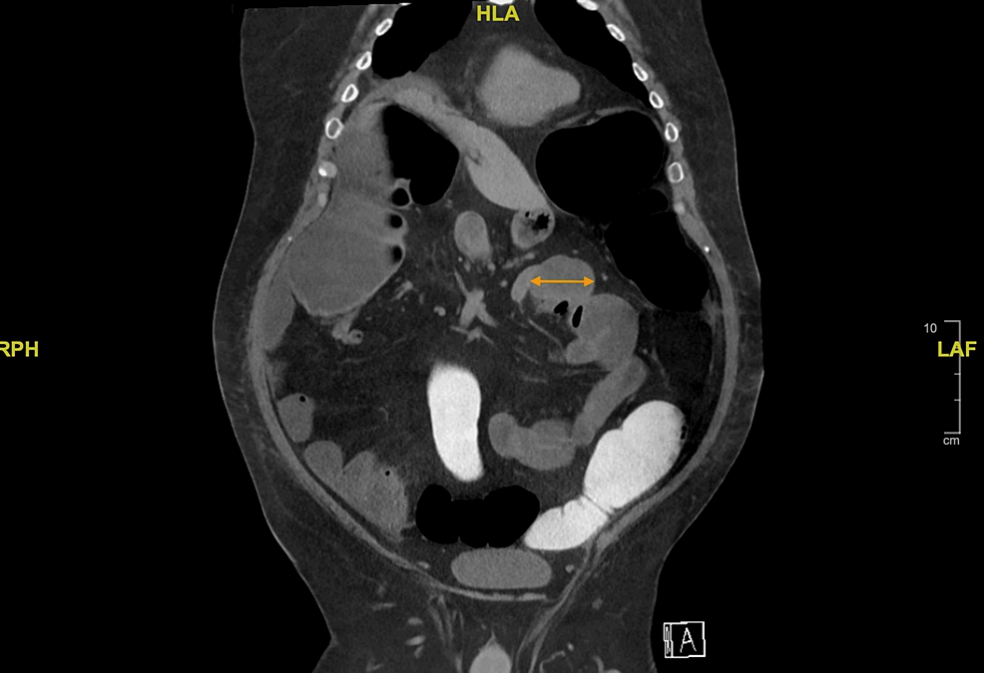
CT abdomen and pelvis with rectal contrast Coronal view of CT abdomen and pelvis with rectal contrast upon first admission showing significantly distended colon and mildly distended small bowel (orange arrows).

**Figure 6:**
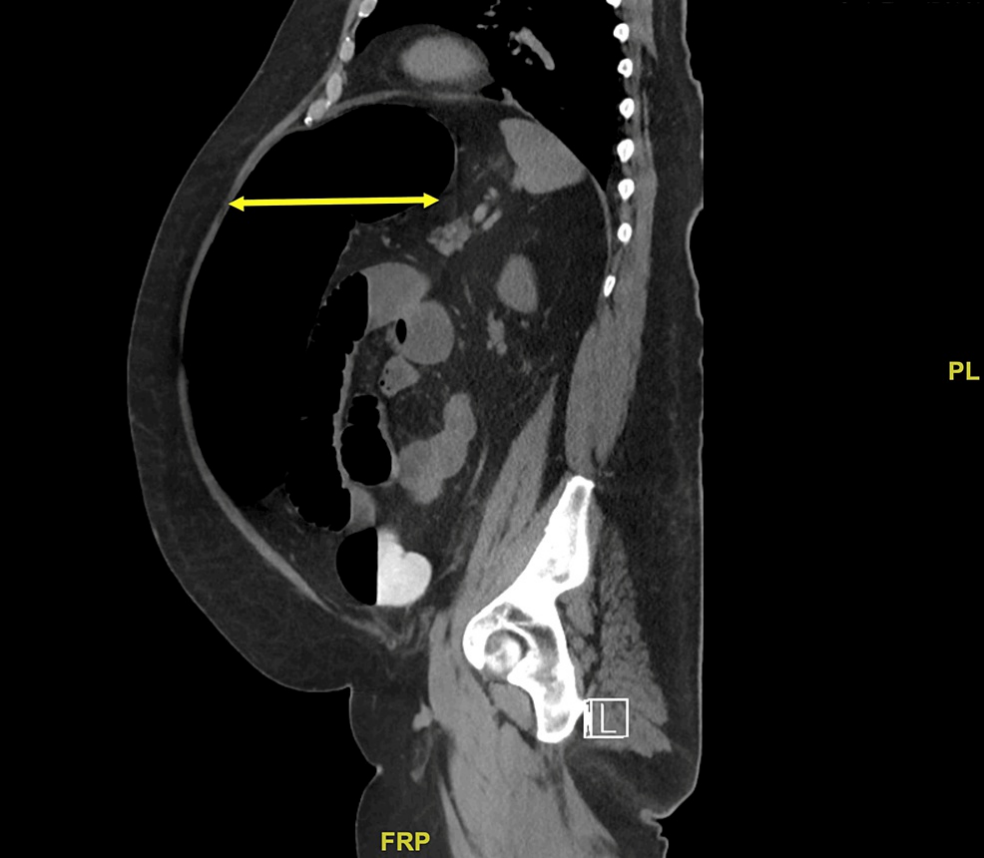
CT abdomen and pelvis with rectal contrast Sagittal view of CT abdomen and pelvis with rectal contrast upon first admission showing distended colon (yellow arrows) with gas.

**Figure 7:**
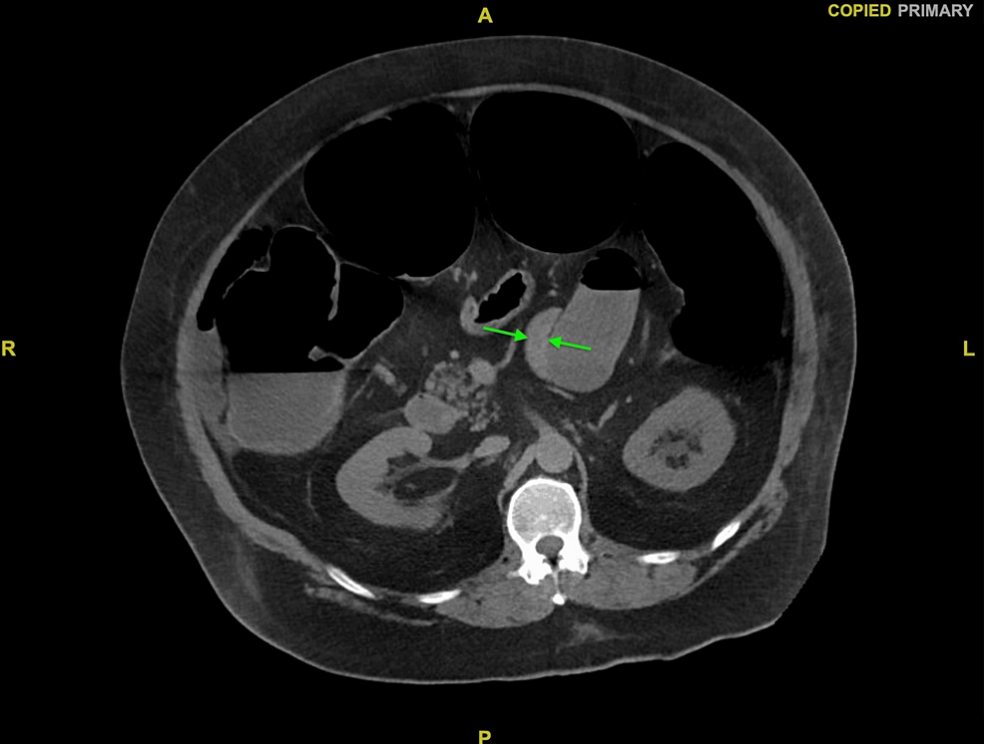
CT abdomen and pelvis with rectal contrast Axial view of CT abdomen and pelvis with rectal contrast upon first admission showing bowel wall thickening and edema (green arrows).

The patient had minimal known risk factors for Ogilvie syndrome aside from hypokalemia upon admission. He had no history of alcohol or tobacco use, cardiovascular disease, recent infection, immobility, or poly-pharmacy. His only home medication was 0.4 mg of tamsulosin daily for BPH. Colonic dilation persisted despite conservative treatment, including electrolyte replacement, nil per os (NPO), and stool softener administration. Due to extensive colonic dilation and a lack of symptom resolution with conservative treatment, the patient was considered for a subtotal colectomy. However, CT imaging showed thickening of the colonic and small bowel walls (Figures 5, 6, 7), concerning potential complications such as anastomotic leakage and poor wound healing if surgery was performed at that time. He instead underwent a colonoscopy with rectal tube placement for symptom resolution. Upon discharge, the patient was told to follow up as an outpatient for further surgical evaluation; however, he was unable to follow up due to socioeconomic barriers such as living in a rural location. Months after his first admission, he was admitted again within 48 hours of another attempt at beef consumption. His second hospital course presented similarly to the first, and he was diagnosed with recurrent Ogilvie syndrome once again, requiring colonoscopy and rectal tube placement for symptom resolution. Before discharge, he was scheduled for surgical intervention three months later.

The patient underwent a subtotal colectomy with ileocolic anastomosis three months after his last admission. He was intubated and sedated, and he was placed in the modified lithotomy position in Yellofin stirrups. An IV antibiotic was given. The rectum was irrigated with a povidone-iodine (betadine) solution using a large Malecot drain. Cystoscopy and bilateral ureteral stent placement were performed after the perianal area was prepped. The sigmoid colon was noted in the pelvic area with adhesions. Multiple other robotic trochars were placed in the abdominal cavity, and the robot was docked. Lysis of adhesions was performed. Adhesions were present between the sigmoid colon and the abdominal wall. The right transverse colon and descending colon were both mobilized. The hepatic and splenic flexures were mobilized. The anastomosis was created in a stable fashion using the robotic blue load 60 mm stapler, and the common enterotomy was closed with a 3-0 polydioxanone (PDS) x2 suture, and the specimen was removed through a 4 cm incision protected with the wound protector. Pathological examination of the colon later revealed a grade I, well-differentiated neuroendocrine carcinoid tumor <1 cm confined to the tip of the appendix with negative margins.

The patient advanced his diet and was eventually able to tolerate soft foods upon discharge within 48 hours post-op. No further complications occurred during his hospital course, and he was discharged for outpatient follow-up. At two weeks post-op, he presented to the clinic due to redness and local tenderness around his wound. An infected midline abdominal hematoma was removed via drainage in the clinic. He subsequently completed a course of metronidazole and ciprofloxacin with complete resolution. He was compliant with abdominal binder use for an umbilical hernia. Additionally, he described improvement in symptoms of bloating and constipation prior to surgery. He tolerated oral intake and remained on a strict diet without products containing AGS. He denied hematochezia, melena, abdominal pain, nausea, or vomiting; however, he was having diarrhea about six to seven times daily, likely due to his surgery rather than a history of AGS, which previously caused constipation. 

**Figure 8:**
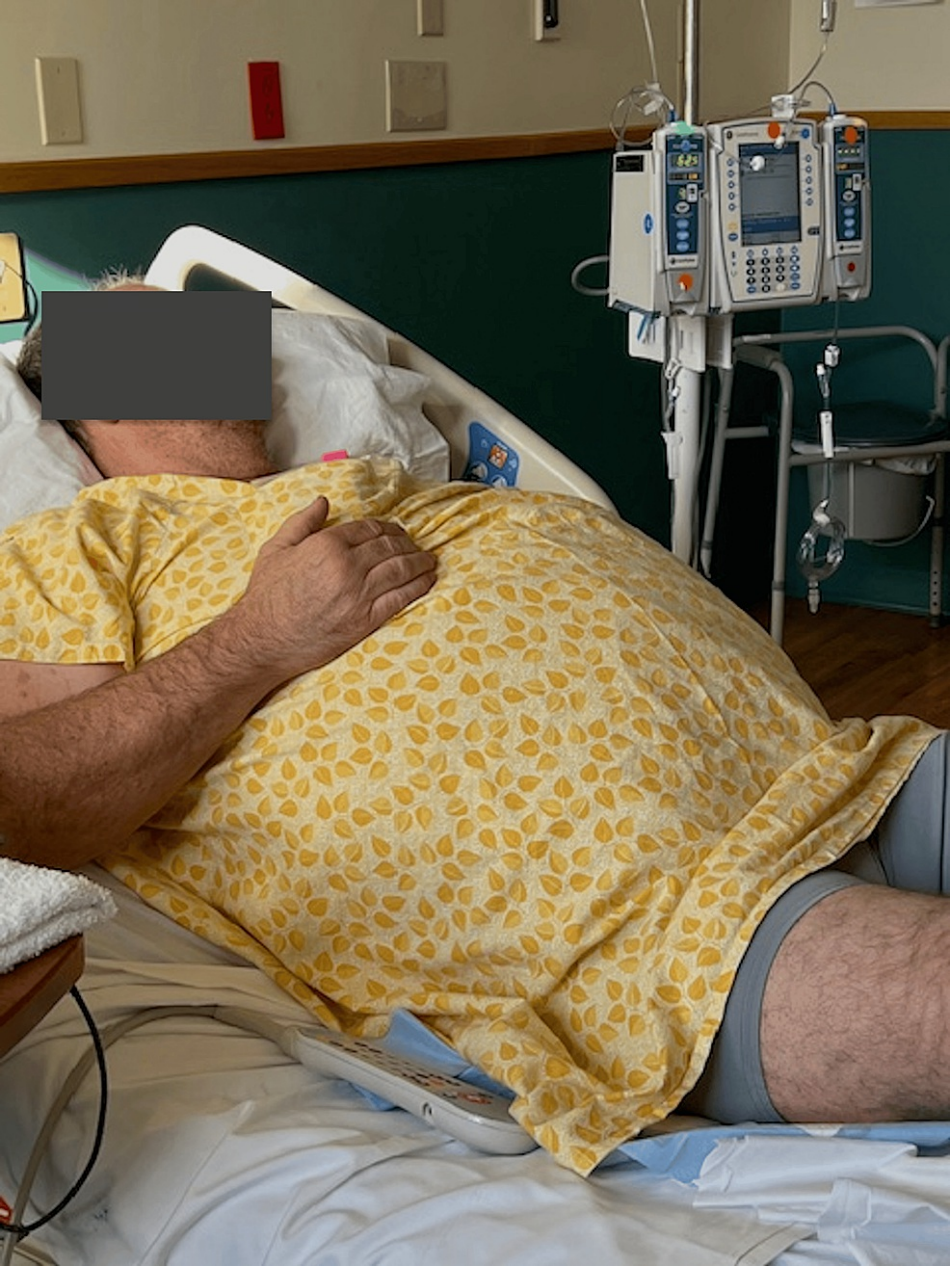
Patient on first admission with Ogilvie syndrome The patient provided this image in order to demonstrate his significant abdominal distention on first admission prior to colonoscopy with rectal tube placement. The patient approved of the use of the image in this case report.

**Figure 9:**
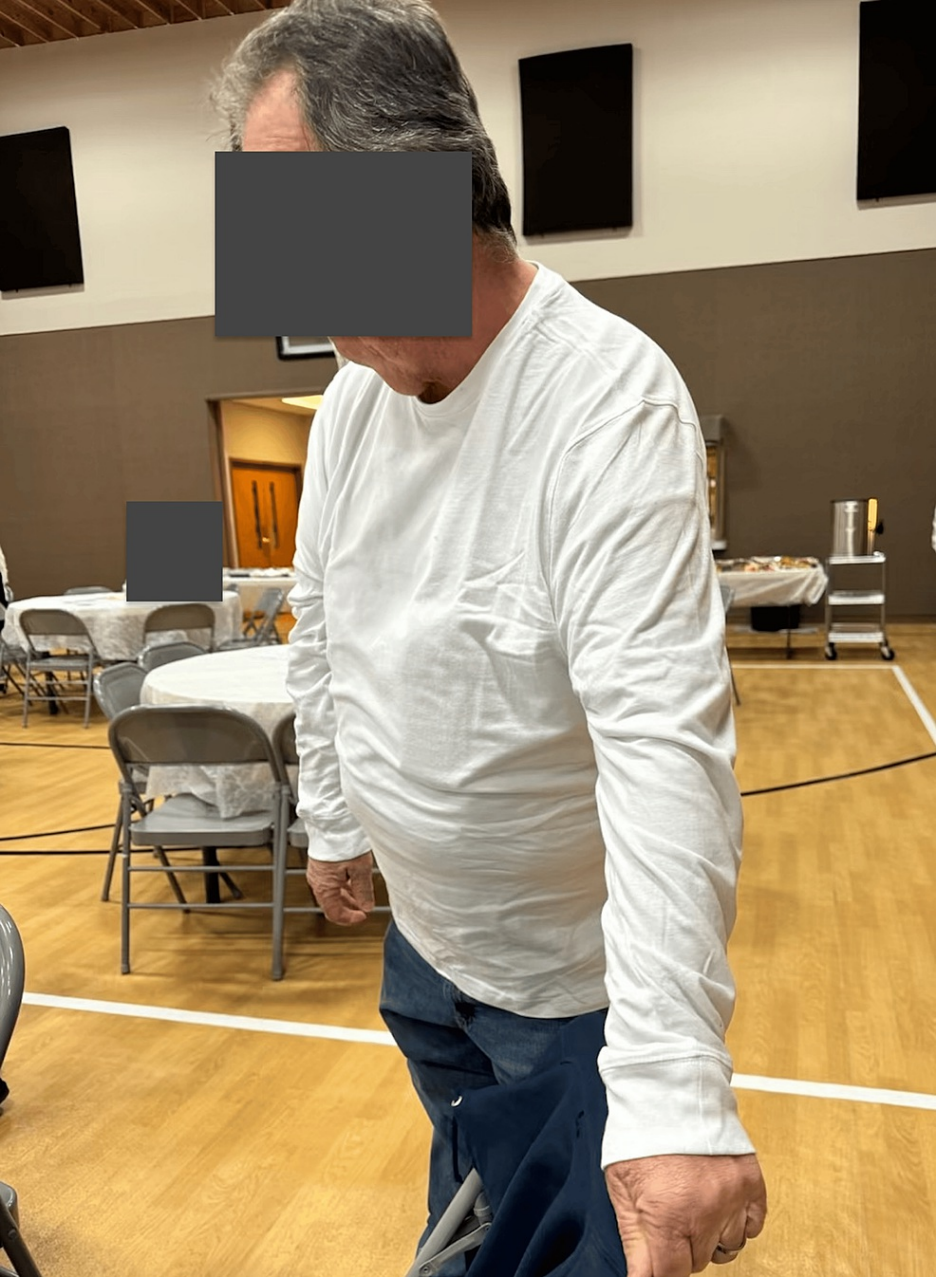
Patient two months post-robotic subtotal colectomy with ileocolic anastomosis The patient provided this image in order to demonstrate improvement in abdominal distention from the first admission, which occurred about two months after surgery. The patient approved of the use of the image in this case report.

At approximately six weeks post-op, the patient reported significant improvement in previous symptoms of constipation and distention. Due to the systemic nature of AGS, he was counseled to avoid alpha-gal consumption without attempting reintroduction, and he endorsed adherence to an alpha-gal-free diet. Additionally, he reported improvements in diarrhea, physical comfort, and overall quality of life. Figures 8, 9 highlight the noticeable reduction in the patient's abdominal circumference from initial hospital admission to two months after surgical management. We hypothesize that the recurrent Ogilvie syndrome in this case was a manifestation of AGS, and surgical treatment was required for prevention of recurrence; however, the exact cause of the patient's Ogilvie syndrome and eventual improvement of constipation and bloating remain unclear.

## Discussion

AGS is diagnosed clinically with supportive evidence such as elevated alpha-gal IgE levels and symptom improvement upon adherence to an alpha-gal-free diet. Although allergies are typically confirmed through oral food challenges, the delayed and inconsistent reactions characteristic of AGS render this impractical for routine care, prompting allergists to monitor symptoms during such challenges in a clinical setting [10].

Management of AGS currently revolves around dietary modification, with patients instructed to avoid mammalian meats and related products while opting for alternatives such as fish, seafood, poultry, and non-mammalian-derived foods [1]. Currently, there is no evidence that AGS patients can “outgrow” their alpha-gal allergy and reintroduce mammalian meat into their diet without symptoms. However, some findings indicate that AGS individuals who actively avoid tick bites demonstrate a reduction in specific alpha-gal IgE levels and symptom severity associated with alpha-gal consumption [11]. Despite claims of effectiveness, there is insufficient evidence to support acupuncture or other interventional methods as a treatment for AGS [12]. Collaborative efforts between gastroenterologists, dietitians, surgeons, and other healthcare professionals are essential in managing and increasing awareness of this condition.

We have high suspicion that AGS played a significant role in the patient's presentation of recurrent Ogilvie syndrome due to the timing of his beef consumption prior to multiple admissions. Our case underscores the importance of recognizing the diverse clinical manifestations of AGS beyond typical allergic reactions. Currently, there are no documented cases of AGS linked to Ogilvie syndrome. However, we cannot conclude that AGS was the direct cause of his symptoms, including those of baseline constipation and abdominal distention. Additionally, the pathological finding of a low-grade appendiceal neuroendocrine tumor (NET) obscures the possible etiology of the patient's acute symptoms. According to research, NETs may present with symptoms such as diarrhea, abdominal pain, and constipation [13]. In this case, the patient reported improvement in symptoms of constipation and abdominal distention after the surgical removal of the tumor. However, there are no reports of NETs associated with Ogilvie syndrome, and the patient was asymptomatic prior to his diagnosis of AGS. Literature supports that grade 1 appendiceal NETs are often asymptomatic unless metastasis occurs, and the majority are diagnosed as incidental findings from appendectomies or other abdominal procedures [14]. More severe manifestations of appendiceal NETs can mimic acute appendicitis and right lower quadrant tenderness, which our patient did not have. On CT imaging, there was no evidence of an abnormality in the appendix, likely due to its small size, and no evidence of metastasis. Due to the size of <1 cm and the low-grade nature of his NET, it was likely an incidental finding that did not cause acute symptoms. 

While we show that surgical intervention may be considered in select cases of AGS, it is crucial to weigh the risks and benefits of surgical management, considering factors such as symptom severity, frequency, and overall patient health status. According to a study by Joechle et al., conservative and endoscopic management fails in about one-third of patients diagnosed with Ogilvie syndrome, and a cut-off diameter ≥ 11 cm may be an adequate parameter to consider surgical therapy [15]. Subtotal colectomy carries inherent risks, including postoperative complications such as anastomotic leakage, infection, and long-term gastrointestinal dysfunction; however, our case showed notable improvement in baseline gastrointestinal symptoms, highlighting the potential utility of managing carefully selected AGS patients with surgery. Exploring whether the symptom improvement stemmed from adherence to a diet or the efficacy of surgical interventions may warrant further investigation. With the rising prevalence of AGS and its potentially life-threatening consequences, there is a need for further research to educate providers and develop more robust diagnostic and treatment methods to improve patient outcomes.

## Conclusions

This is the first case report of recurrent Ogilvie syndrome as a possible manifestation of AGS requiring surgical management for symptom resolution. Healthcare providers should be educated on AGS and consider it a differential diagnosis due to its increasing prevalence and wide range of clinical presentations. Surgical management may be a viable option for complications of AGS and could be considered in cases with rare manifestations.

## References

[1] Commins SP (2020). Diagnosis & management of alpha-gal syndrome: lessons from 2,500 patients. Expert Rev Clin Immunol.

[2] Mitchell CL, Lin FC, Vaughn M, Apperson CS, Meshnick SR, Commins SP (2020). Association between lone star tick bites and increased alpha-gal sensitization: evidence from a prospective cohort of outdoor workers. Parasit Vectors.

[3] Patel C, Iweala OI (2020). 'Doc, will I ever eat steak again?': diagnosis and management of alpha-gal syndrome. Curr Opin Pediatr.

[4] Román-Carrasco P, Hemmer W, Cabezas-Cruz A, Hodžić A, de la Fuente J, Swoboda I (2021). The α-gal syndrome and potential mechanisms. Front Allergy.

[5] Epelboin L, Roche F, Dueymes M (2021). Allergy to mammalian meat linked to alpha-gal syndrome potentially after tick bite in the Amazon: a case series. Am J Trop Med Hyg.

[6] Platts-Mills TA, Li RC, Keshavarz B, Smith AR, Wilson JM (2020). Diagnosis and management of patients with the α-gal syndrome. J Allergy Clin Immunol Pract.

[7] Thompson JM, Carpenter A, Kersh GJ, Wachs T, Commins SP, Salzer JS (2023). Geographic distribution of suspected alpha-gal syndrome cases - United States, January 2017-December 2022. MMWR Morb Mortal Wkly Rep.

[8] Conner S, Nassereddin A, Mitchell C (2024). Ogilvie Syndrome. StatPearls [Internet.

[9] Vanek VW, Al-Salti M (1986). Acute pseudo-obstruction of the colon (Ogilvie's syndrome). An analysis of 400 cases. Dis Colon Rectum.

[10] McGill SK, Hashash JG, Platts-Mills TA (2023). Aga clinical practice update on alpha-gal syndrome for the GI clinician: commentary. Clin Gastroenterol Hepatol.

[11] Kim MS, Straesser MD, Keshavarz B, Workman L, McGowan EC, Platts-Mills TA, Wilson JM (2020). IgE to galactose-α-1,3-galactose wanes over time in patients who avoid tick bites. J Allergy Clin Immunol Pract.

[12] Bernal M, Huecker M, Shreffler J, Mittel O, Mittel J, Soliman N (2021). Successful treatment for alpha gal mammal product allergy using auricular acupuncture: a case series. Med Acupunct.

[13] Deans GT, Spence RA (1995). Neoplastic lesions of the appendix. Br J Surg.

[14] Griniatsos J, Michail O (2010). Appendiceal neuroendocrine tumors: recent insights and clinical implications. World J Gastrointest Oncol.

[15] Joechle K, Guenzle J, Utzolino S, Fichtner-Feigl S, Kousoulas L (2022). Ogilvie's syndrome-is there a cutoff diameter to proceed with upfront surgery?. Langenbecks Arch Surg.

